# Using unsupervised capsule neural network reveal spatial representations in the human brain

**DOI:** 10.1002/hbm.26573

**Published:** 2024-03-27

**Authors:** Gongshu Wang, Ning Jiang, Tiantian Liu, Li Wang, Dingjie Suo, Duanduan Chen, Shintaro Funahashi, Tianyi Yan

**Affiliations:** ^1^ School of Medical Technology Beijing Institute of Technology Beijing China; ^2^ Advanced Research Institute for Multidisciplinary Science Beijing Institute of Technology Beijing China; ^3^ Department of Cognitive and Behavioral Sciences, Graduate School of Human and Environmental Science Kyoto University Kyoto Japan; ^4^ Kokoro Research Center Kyoto University Kyoto Japan

**Keywords:** brain encoding, brain model, fMRI, spatial working memory

## Abstract

Humans can extract high‐level spatial features from visual signals, but spatial representations in the brain are complex and remain unclear. The unsupervised capsule neural network (U‐CapsNet) is sensitive to the spatial location and relationship of the object, contains a special recurrent mechanism and uses a self‐supervised generation strategy to represent images, which is similar to the computational principle in the human brain. Therefore, we hypothesized that U‐CapsNet can help us understand how the human brain processes spatial information. First, brain activities were studied using functional magnetic resonance imaging during spatial working memory in which participants had to remember the locations of circles for a short time. Then, U‐CapsNet served as a computational model of the brain to perform tasks that are identical to those performed by humans. Finally, the representational models were used to compare the U‐CapsNet with the brain. The results showed that some human‐defined spatial features naturally emerged in the latent space of U‐CapsNet. Moreover, representations in U‐CapsNet captured the response structure of two types of brain regions during different activity patterns, as well as important factors associated with human behavior. Together, our study not only provides a computationally feasible framework for modeling how the human brain encodes spatial features but also provides insights into the representational format and goals of the human brain.

## Graphical abstract

We assume that U‐CapsNet has higher biological plausibility than the traditional DNNs in modeling the brain to encode spatial information. Our results suggest that a synergy between U‐CapsNet and spatial cognitive neuroscience offers the promising prospect for understanding the internal representations of intelligent systems and the functional mechanisms of the brain's visual system.

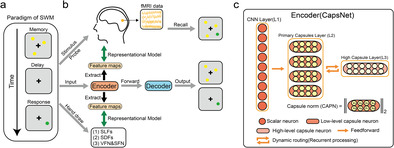

## 1 INTRODUCTION

Deep neural networks (DNNs) provide powerful computational models for deep feature extraction from visual signal (LeCun et al., 2015). In the last decade, several studies have explored the connection between brain neural and DNN representations by training DNNs to perform tasks similar to those performed by primates, thereby improving the understanding of natural visual perception. Khaligh‐Razavi & Kriegeskorte (2014) illustrate that DNN representations explain activity in inferior temporal cortex using functional magnetic resonance imaging (fMRI). Seeliger et al. (2018) show that DNN representations are expressed in space and time across the cortical surface using magnetoencephalogram (MEG). Wen et al. (2018) show that DNNs can elucidate visual cortex activity in response to movie stimuli using fMRI. Chang et al. (2021) indicate that DNNs possess the capability to explicate cortical activation in face recognition tasks using fMRI and electroencephalogram (EEG). These findings suggest that DNNs use representations similar to those of the brain (Mehrer et al., 2021) and achieve promising results in modeling brain responses to visual stimuli (Palazzo et al., 2021; Shen et al., 2019). Moreover, recent works have used DNNs to accurately model complex brain cognition, such as picture imagery, esthetic value, and decision‐making ability (Cross et al., 2021; Iigaya et al., 2020; Xie et al., 2020), suggesting the potential of DNNs to uncover brain higher cognitive mechanisms (Saxe et al., 2021).

Spatial working memory (SWM), a core component of cognitive functions, is not a simple category recognition, and observers need to compute ensemble statistics about a display accurately. Neuroimaging studies have demonstrated that SWM function relies on the prefrontal and parietal lobes and dorsal stream areas (Ester et al., 2015; Silk et al., 2010). Among them, dorsal stream areas play an important role in extracting spatial features (Ayzenberg & Behrmann, 2022). However, how the human brain transforms images into high‐level spatial features remains unclear. Therefore, we aimed to develop a biologically plausible computational model to understand the computation and representation during SWM.

Most previous studies utilized supervised feedforward convolutional neural networks (SFF‐CNNs), such as VGG, AlexNet, ResNet, and so forth, to represent the visual system, but these networks differ from the brain in many aspects (Pulvermüller et al., 2021). On the one hand, SFF‐CNNs lack the brain‐like recurrent processing (for instance, upon receiving information from “Neuron A,” “Neuron B” may provide feedback and ask for clarification or additional details to enhance the quality of the information) in their built‐in structure (Kietzmann et al., 2019), perform differently from humans in crucial psychophysical tasks and are vulnerable to disruptions (Doerig et al., 2019; Geirhos et al., 2018). In this case, SFF‐CNNs mainly focus on local texture and shape (Baker et al., 2018), while humans perform global contextual calculations (Doerig et al., 2019). It was suggested that altering the local properties of an object causes SFF‐CNNs to misidentify items, but humans can still correctly identify the object according to its global context.

On the other hand, supervised models are not directly optimized to fit neural responses but rather to learn label‐related representations (Yamins & DiCarlo, 2016). These models may provide an incorrect explanation of how cortical neural representations are learned in the first place (Higgins et al., 2021; Zhuang et al., 2021). Additionally, some studies provide evidence that the visual system leverages unsupervised learning to encode the semantically meaningful embeddings of sensory signals (Lindsay, 2021; Seung & Lee, 2000).

Therefore, new DNN models are needed to capture the pertinent neural mechanisms (Rajalingham et al., 2018) more precisely. The capsule neural network (CapsNet) is a recently proposed DNN framework. It uses vector neuron—capsule (CAP), as the neuron unit and includes a dynamic routing mechanism (Sabour et al., 2017). A CAP represents the various attributes of a specific entity existing in the image, such as pose, deformation, and texture. Dynamic routing integrates important information from low‐level CAPs into high‐level CAPs through a feedback mechanism. The validity of the dynamic routing is also supported by invariant pattern recognition in the visual cortex. Recently, psychophysical evidence has shown that CapsNet naturally reproduces human behavior in a visual grouping and segmentation task (Doerig et al., 2020).

Recent studies have suggested that unsupervised methods predict neural activity in ventral (Higgins et al., 2021; Zhuang et al., 2021) and dorsal (Bakhtiari et al., 2021) visual cortical areas with accuracy equal to or greater than the most advanced supervised methods available today. They have focused on the evaluation of variational autoencoders (VAEs) for modeling brain responses to natural video stimulus (Han et al., 2019) and have shown a strong association between the representations encoded by β‐VAEs and those generated by single neurons (Higgins et al., 2021).

**FIGURE 1:**
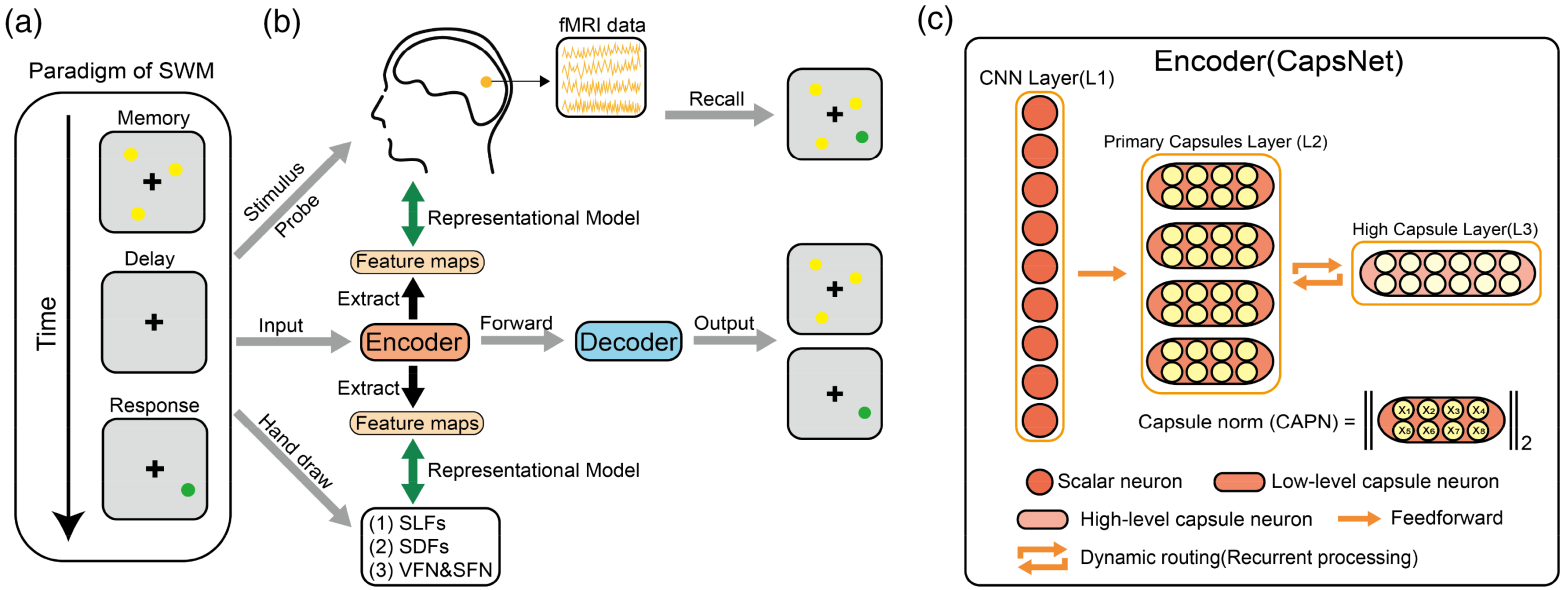
Workflow of the study. (a) Experimental paradigm of spatial working memory (SWM). A trial consisted of three phases: memory, delay, and response. (b) Participants were collected fMRI data while performing SWM tasks (“Stimulus” and “Probe” correspond to yellow and green circles, respectively). The images presented to the participants were then fed into the trained model to obtain the feature maps from each layer in encoder. In addition, some human‐defined functions were used to compute high‐level spatial features in images (see Figure 2a and Section 2). Representational models were used to analyze similarities between brain activation, feature maps in U‐CapsNet, and high‐level spatial features. (c) The architecture of CapsNet, which consists of a CNN layer, a primary capsule layer, and a high capsule layer. ${\left\|∙\right\|}_{2}$ indicates the calculation of the L2‐Norm of a vector.

Inspired by the above insights, we hypothesized that unsupervised learning combined with CapsNet might show more biological feasibility. The workflow of our study is shown in Figure 1. First, we analyzed fMRI data collected from participants performing SWM to characterize patterns of brain activity. Next, we trained an unsupervised CapsNet (U‐CapsNet) serve as a computational model to perform the same SWM tasks as the human brain. Finally, we compared brain activation and representations in U‐CapsNet from multiple perspectives using representational similarity analysis (RSA) and fMRI encoding models. Our objective is to test whether U‐CapsNet is similar to the human brain and has the ability to reveal the form and goals of spatial information representation in the brain.

## 2 MATERIALS AND METHODS

### 2.1 Participants

The neuroimaging dataset was shared by the UCLA Consortium for Neuropsychiatric Phenomics LA5c Study and approved by the UCLA Institutional Review Board (Poldrack et al., 2016). The dataset included 139 subjects who had no relevant medical or psychiatric information, of which 129 subjects of these (females = 61, mean age = 31.43 years, SD = 8.76, range = 21–50) performed SWM tasks functional magnetic resonance imaging (fMRI) collection. No subjects were removed for subjective reasons. The data were obtained via the public database OpenfMRI and were made available under the Public Domain Dedication and License v1.0.

### 2.2 Image acquisition

Whole‐brain imaging data were acquired on one of two 3 T Siemens Trio scanners (Siemens version Syngo MR B15 and B17). Task‐state fMRI data were collected using a T2‐weighted echoplanar imaging (EPI) sequence with the following parameters: repetition time (TR) = 2 s, echo time (TE) = 30 ms, flip angle = 90°, slice thickness = 4 mm, slices = 34, matrix = 64 × 64, and field of view (FOV) = 192 mm. The task‐state fMRI scan lasted approximately 10 min. In addition, a high‐resolution anatomical magnetization‐prepared rapid acquisition with gradient echo (MPRAGE) scan was performed. The parameters for MPRAGE were as follows: TR = 1.9 s, TE = 2.26 ms, matrix = 256 × 256, FOV = 250 mm, sagittal plane, slice thickness = 1 mm, and slices = 176.

### 2.3 Behavioral paradigm and memory performance

During the SWM task, subjects were shown a target array of 1, 3, 5, or 7 yellow circles pseudo‐randomly placed around a central fixation cross. After a delay, subjects were shown a single green circle and had to indicate whether that circle was in the same position as one of the target circles had been. To allow subjects to adequately encode the target array, a relatively long stimulus presentation time of 2 s was used. Decision or selection requirements were kept constant across set sizes to reduce possible effects of set size on response processes. In addition to load, the delay period was manipulated, with delays of 1.5, 3, or 4.5 s. Trial events included a 2‐s target‐array presentation: a 1.5, 3, or 4.5 s delay period; and a 3‐s fixed response interval. A central fixation was visible throughout each of the 48 trials (12 per memory set size, with 4 at each delay length for each memory set). Half the trials were true‐positive, and half were true‐negative. Before starting the in‐scanner task, subjects underwent a supervised instruction and training period outside of the scanner; once inside the scanner, they were again reminded of the instructions. After scanning, the response accuracy (percentage of correct decisions in 48 trials) of each subject was calculated to indicate memory performance. The order and content of the visual stimuli were identical for all participants.

### 2.4 fMRI preprocessing

Task‐state fMRI data were preprocessed using SPM12 as follows: slice time correction; head motion correction with alignment to the minimum outlier in the time series; registration with T1‐weighted image unified segmentation and spatial normalization to the Montreal Neurological Institute (MNI) template; and Gaussian filter smoothing (full‐width‐at‐half‐maximum [FWHM]: 4 mm).

### 2.5 Task activation estimation

Each trial event was divided into the memory stage (where the yellow circle appeared), delay stage, and response stage (where the green circle appeared). This study focused on the activations in the memory stage and the response stage. For each memory stage, task timing (from the appearance to the disappearance of the yellow circle(s)) was convolved with the SPM canonical hemodynamic response function (HRF) and entered into a standard fMRI general linear model (GLM), as well as the nuisance covariates (six motor parameters and their derivatives and mean white matter and mean cerebrospinal fluid signals). For each response stage, task timing (from the appearance to the disappearance of the green circle), was also convolved with the HRF and entered into another GLM, as well as the nuisance covariates. As a result, we obtained two task‐related β series voxelwise from each subject, which represented the activations of the memory and the response stage respectively.

### 2.6 Parcellation of the brain

In RSA, voxels were grouped into regions of interest according to the Brainnetome (BN) Atlas (Fan et al., 2016). This atlas divides the brain into 246 regions covering both cortical and subcortical and groups these 246 regions into 25 sections. This study focused on 132 regions in 13 sections. In encoding model analysis, voxels were grouped into five regions: frontal, ventral (temporal) and dorsal (parietal), early visual cortex, and hippocampus (Figure S1).

### 2.7 Calculation of visual spatial features

We manually calculated some common location and distance features to characterize the spatial distribution and dispersion degree of the circles. These features were (1) the number of circles in each quadrant (every 90 degrees is a quadrant) and (2) the center position of the graph, in X and Y coordinates, surrounded by small circles. (3) The average distance between each circle and the fixation cross. (4) The average distance between each circle and the origin (bottom left corner of the picture). (5) The average distance between the circles (if there is only one circle in the picture, the distance is 0). Spatial location features (SLFs) include (1) and (2), and spatial distance features (SDFs) include (3)–(5). VFN & SFN concatenates the L2‐Norm of principal components (PCs) of the image, L2‐Norm of SLFs, and L2‐Norm of SDFs, representing the abstract features of the image.

### 2.8 Unsupervised capsule neural network model

We designed an unsupervised U‐CapsNet based on an encoder‐decoder architecture to reconstruct input features. The encoder is a simple CapsNet and the decoder is implemented by deconvolution. The CapsNet consists of three layers: convolutional layers, primary capsule layer, and higher capsule layer. A fully connected layer links the encoder and decoder. The decoder consists of three sequential deconvolution layers. The parameter settings of the network are shown in Table S1. The dynamic routing process occurs between the primary capsule layer and the higher capsule layer. For more details and principles in the CapsNet refer to Sabour et al. (2017).

The vector (capsule) $u_{i}$ is transformed to form a predicted vector ${\hat{u}}_{j|i},$ which predicts the output of the capsule 𝑖 to higher capsule 𝑗.

(1) $${\hat{u}}_{i|j}=W_{\mathrm{ij}}u_{i},$$

$W_{\mathrm{ij}}$ denotes the weight matrix, which is trainable. The higher capsule 𝑗 receives input from the lower capsules through a weighted summation of their predicted vectors, as follows:

(2) $$s_{j}=\sum_{i\in I}c_{\mathrm{ij}}{\hat{u}}_{j|i,}$$

where $c_{\mathrm{ij}}$ indicates the routing coefficient from the lower capsule 𝑖 to the higher level capsule. 𝐼 is the set of all lower capsules. The $c_{\mathrm{ij}}$ is calculated by a softmax function as follows:

(3) $$c_{\mathrm{ij}}=\exp\left(b_{\mathrm{ij}}\right)/\sum_{i\in I}\exp\left(b_{\mathrm{ij}}\right),$$

where $b_{\mathrm{ij}}$ is a logarithmic prior probability that capsule 𝑖 is coupled to capsule 𝑗, which is iteratively updated. J is the set of all capsules in the higher layer.

(4) $$b_{\mathrm{ij}}\leftarrow b_{\mathrm{ij}}+{\hat{u}}_{j|i}∙v_{j,}$$

where $v_{j}$ is the output vector of capsule 𝑗 and obtained by a non‐linear “squashing” function as follow:

(5) $$v_{j}=\frac{{\left\|s_{j}\right\|}^{2}}{1+{\left\|s_{j}\right\|}^{2}}\frac{s_{j}}{\left\|s_{j}\right\|}$$

There were only 48 pairs of images in the SWM task, and we created an additional 400 images with a black background and yellow circles (the number of yellow circles in each image was 1, 3, 5, or 7, and there were 100 images of each type) to expand the number of samples. The 400 created images were the training set. Images were resized to 30 × 30 and normalized before running the model. The training consisted of 3000 epochs, using one GPU (Nvidia Tesla P100). The loss function is the mean absolute error (MAE) between the original image and the generated image. In each iteration, the model parameters were updated using an adaptive moment estimation (ADAM) optimization algorithm. The learning rate is 1 × 10^−4^. After training, the images used to stimulate participants were input into the trained model generating feature maps of each layer in U‐CapsNet. The feature map of Layer 1 was 256 × 22 × 22, the feature map of Layer 2 was 64 × 7 × 7 × 10 (3136 CAPs), and the feature map of Layer 3 was 1 × 40 (1 CAP). Of note, the basic unit in Layer 2 was CAP which was a vector of dimension 10, and we also calculate the L2‐Norm (length of the vector) for each CAP, resulting in a 67 × 7 × 7 feature map.

### 2.9 Control models

To construct a control model for the representation of basic visual features, the image pixels were linearly projected into low dimensions using principal components analysis (PCA). The transformation matrix was estimated using the images in the training set.

To test whether the self‐supervised generation target outperforms the supervised classification target in the modeling brain, we trained two supervised models and determined the image label based on the number of circles present. One of these was an SFF‐CNN consisting of three convolution layers and two linear projection layers. The features in the third convolution layer were used as the control model. Another is a supervised CapsNet (S‐CapsNet), which added two linear projection layers to the encoder of the U‐CapsNet. The CAP in the higher capsule layer was used as the control model. Their training set consisted of 3000 epochs, and the learning rate was 1 × 10^−4^. Cross entropy represented the loss function.

To test whether the self‐supervised generation target combined with CapsNet performs better in modeling the brain, we trained another self‐supervised model—VAE (Kingma & Welling, 2013) whose architecture is similar to that of U‐CapsNet. Its encoder consists of three convolution layers, followed by a linear projection layer to output the set of the “mean” and “log‐variance” parameters for the latent space. Its decoder consists of three deconvolution layers. Each stimulus image was input into the trained model and mapped to the latent distribution, which included 40 means and 40 log variances. The means were then used as a feature set for the control model. The training consisted of 3000 epochs. The learning rate is 1 × 10^−4^.

The parameter settings for the control methods are shown in Tables S2–S4. Note that for a fair comparison, we set the number of layers of the control DNN models (SFF‐CNN, S‐CapsNet, and VAE) similar to that of U‐CapsNet.

### 2.10 Representational similarity analysis

RSA was used to examine the consistency between computational models and brain activity. Firstly, representational dissimilarity matrices (RDMs) were constructed at the image level for three layers in U‐CapsNet, pixel space, SLFs, SDFs, VFN & SFN, control models, and fMRI data by computing pairwise comparisons across images. For pixel space, the image was resized to 30 × 30, and then the 30 × 30 × 3 tensor was reshaped to a 2700 vector. For fMRI data, voxelwise activation in a region was arranged into a vector, and RDMs for every region were constructed. Correlation distance was used as a distance metric for fMRI data. Euclidean distance (ED) was used for hand‐drawn features and computational models. Then, the Spearman correlation was used to analyze the relationship between RDMs of hand‐drawn features and computational models (the RDMs of hand‐drawn features do not follow a normal distribution). Pearson correlation was used to analyze relationships between RDMs of computational models and fMRI data (the RDMs of computational models and fMRI data follow a normal distribution), which increases sensitivity to differences among representational models and facilitates comparison of model performance (Diedrichsen & Kriegeskorte, 2017; Ejaz et al., 2015).

The noise in the brain activation has limited the amount of dissimilarity variance that can be explained by a model RDM. Therefore, estimating the noise ceiling help to indicate how much variance in a brain RDM was likely to be explained by a desired model RDM. To estimate an appropriate noise ceiling, we calculated the average correlation of each individual RDM with the group mean, regarding the group mean as a proxy for the ideal model. As a lower bound, each individual RDM was also correlated with the group mean from which this individual was excluded (Ejaz et al., 2015).

### 2.11 Encoding model

To map latent representations in U‐CapsNet to brain activation voxelwise, we constructed DNN‐based encoding models. When encoding voxel activation at the memory stage, PCA was used to reduce the feature dimension of each U‐CapsNet layer and control model. Then the PCs that retained 99% of the variance were applied as regressors to predict the task‐evoked activation of each voxel by a linear regression model. Of note, when encoding voxel activation at the response stage, we first concatenated the features of two images (the yellow circle image and the corresponding green circle image) and then carried out the above operation.

We assessed regression model performance in predicting voxel responses in held‐out data using leave‐one‐out‐cross‐validation (LOOCV). Regression weights were estimated by ordinary least squares multiple linear regression. Accuracy was estimated using Pearson correlation between predicted and actual activation series.

### 2.12 Statistical analysis

To test the significance of RSAs, we performed permutation tests for each model. Correlation analysis was performed after the RDMs were shuffled. Then, we tested whether the correlation score was greater than the maximum correlation in the permutation test distribution (1000 permutations, threshold *p* < .05). For RDMs of fMRI data, the permutation test was performed in each brain region in each of the 129 subjects.

Mediation analysis was used to analyze the relationship among spatial\abstract features, computational models, and fMRI. Spatial\abstract features as independent variables (IV), model representation as mediator (Med), brain activity as dependent variable (DV). First, RDMs of brain activation were averaged across regions, and then averaged across subjects. The linear regression models are then used to estimate the parameters a, b, and c, as well as the significance of the model, as follows:

(6) Med=a∙IV+e1,

(7) DV=b∙Med+c∙IV+e2,

where e1 and e2 denote the residuals. Finally, the Sobel test is used to analyze the relationship between a, b, and c to determine if there is a mediating effect between the variables (threshold *p* < .001).

Two‐side Welsch's *t*‐test was used for all pairwise model comparisons. Pearson correlation analysis was used to estimate the similarity between neuroimaging variables and behavioral variables. We used FDR to perform multiple comparison corrections.

## 3 RESULTS

### 3.1 U‐CapsNet focuses on spatial features

**FIGURE 2:**
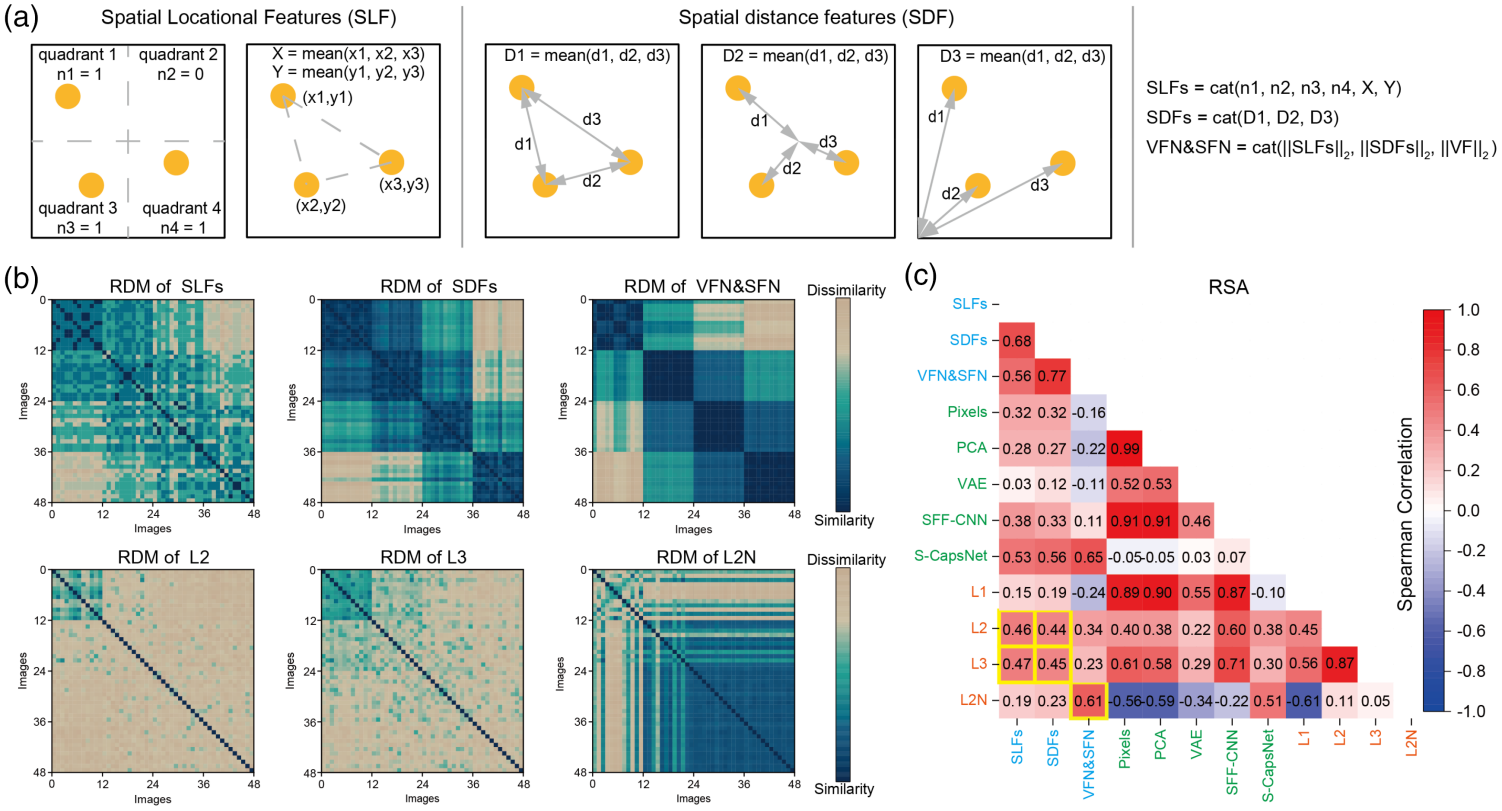
Representational similarity analysis (RSA) between computational models and spatial features. (a) Definition of high‐level spatial features (See Section 2 for details). $\mathrm{cat}\left(∙\right)$ indicates the concatenation operator. ${\left\|∙\right\|}_{2}$ indicates the calculation of the L2‐Norm of a vector. VF represents low‐level visual features, obtained by performing the principal component analysis (PCA) on the image. (b) Representational dissimilarity matrices (RDMs) (representational geometry) of spatial features and feature maps in U‐CapsNet (This study focused on capsule layers, so we didn't show the RDM of Layer 1 of U‐CapsNet). (c) Correlation between RDMs of computational models and spatial features. Positive values (red) indicate similarity, negative values (blue) indicate dissimilarity. Orange font indicates layers in U‐CapsNet. Green font indicates the computational models for the comparison. Blue font indicates manually computed spatial features. Pixels: image is reshaped as a vector, PCA: PCs of the image, VAE: “means” in latent space, SFF‐CNN: features in the third convolution layer, S‐CapsNet: CAP in the higher capsule layer, L1: neurons in the Layer 1 of U‐CapsNet, L2: CAPs in Layer 2 of U‐CapsNet, L3: higher CAP in Layer 3 of U‐CapsNet, and L2N: CAPNs in Layer 2 of U‐CapsNet. SLFs and SDFs were more similar to S‐CapsNet, L2, and L3. VFN & SFN were more similar to S‐CapsNet and L2N. Pixels were more similar with PCA, SFF‐CNN, and L1.

We first drew the spatial features of the images, including spatial location features (SLFs) and spatial distance features (SDFs) (Figure 2a). These features are subjectively defined by human beings, so we regarded them as high‐level spatial features. Previous studies also used manual features or subjective judgments to represent images (Cross et al., 2021; Iigaya et al., 2020). Then we extracted the feature map in each layer, meaning complex nonlinear changed image. Finally, we used RSA, a useful method to compare the representation geometry (Figure 2b) between models (Kriegeskorte et al., 2008; Kriegeskorte & Kievit, 2013), to explore whether the U‐CapsNet incorporates these high‐level spatial features. The same analysis was also performed for the control models to demonstrate the superiority of U‐CapsNet and the positive effects of each of its components. In Figure 2c, the CNN layer (Layer 1) was most similar to the pixel space (*r* = 0.89) and PCs of images (*r* = 0.90) but less similar to the SLFs (*r* = 0.15) and SDFs (*r* = 0.19). The results of VAE and SFF‐CNN were similar to those of Layer 1, indicating that the scalar neurons and feedforward architecture were insensitive to high‐level spatial features and are more concerned with low‐level visual features. In contrast, the primary capsule layer (Layer 2) and the high capsule layer (Layer 3) have high similarity with the SLFs (*r* = 0.46 and 0.47) and SDFs (*r* = 0.44 and 0.45), whose performance is only lower than that of S‐CapsNet. We suspected that the norm/length of CAP could be regarded as the further compression or integration of representations. To verify our hypothesis, we calculated the L2‐Norm of every capsule (CAPN) in Layer 2, and estimate the abstract features of the images using the VFN & SFN (Figure 2a, see Section 2 for detail). The results indicated that the CAPNs in Layer 2 were highly similar to the VFN & SFN (*r* = 0.61), less similar to the SLFs (*r* = 0.19) and SDFs (*r* = 0.23) and dissimilar to the pixel space (*r* = −0.56) and the PCs of images (*r* = −0.59). Therefore, the CAPNs capture high‐level and abstract features and filter out low‐level visual features, which were also of great importance. Together, these findings demonstrated that vector neurons, recurrent processing and self‐supervised generation were beneficial to capture spatial features and that representations in Layer 2 of U‐CapsNet captured multiple levels of image features, which could condense specific visual and spatial features into abstract features or be used to retrieve specific visual and spatial features from abstract features.

### 3.2 Features in U‐CapsNet explain brain activation during the memory stage

**FIGURE 3:**
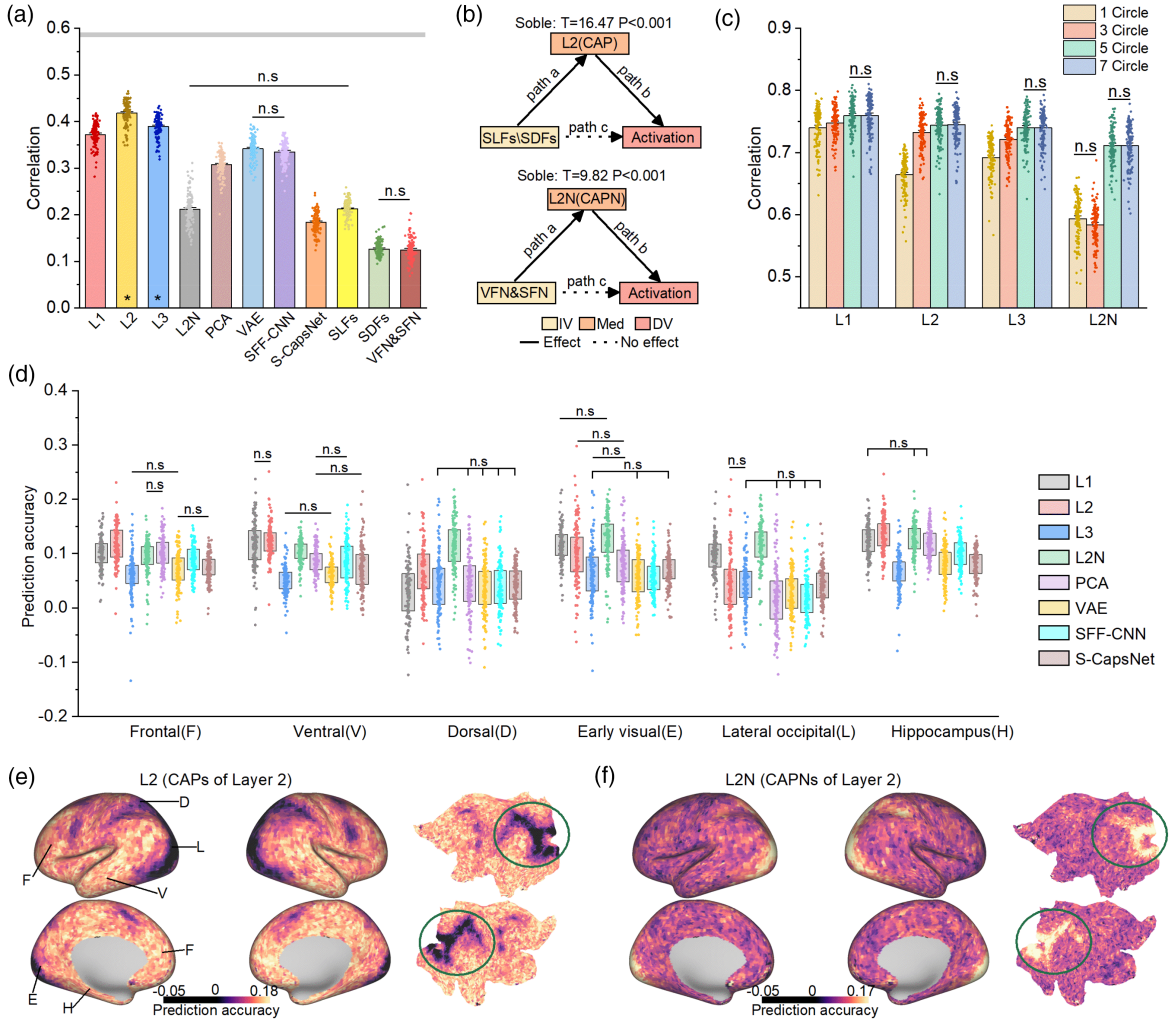
Representational similarity analysis (RSA) between features in U‐CapsNet and brain activation during the memory stage. (a) RSA for each model. Asterisks (*) at the bottom indicates significance in all ROIs of all subjects (1000 permutation tests, *p* < .05). The gray area indicates the noise ceiling. (b) RSA for U‐CapsNet under different conditions. (c) Mediation analysis among representational dissimilarity matrices (RDMs) of spatial/abstract features, CAPs/CAPNs, and brain activation. IV: independent variable, Med: mediator, DV: dependent variable. (d) Predicting voxelwise activation using representations of each computational model. Accuracy is measured by the correlation between actual and predicted activation and is averaged across voxels within each region. The data in the box range from 25% to 75%. (e) and (f) Visualization of prediction accuracy using CAPs and CAPNs in Layer 2 (averaged across all subjects). The green circle approximately covers the dorsal visual stream area. Note that the visualization is in the surface space, and only a portion of the hippocampus was shown. The error bars in (a) and (b) indicate the standard error (SE). (n.s) in (a), (b), and (d) indicates no significant differences between pairs of groups.

To characterize neural mechanisms during SWM, we used RSA to explore whether U‐CapsNet can account for multivoxel representations (Kriegeskorte et al., 2008; Kriegeskorte & Kievit, 2013). First, we established RDM for each brain region in each of the 129 subjects and then calculated the correlation between RDMs of computational models and brain activation during the memory stage. Figure 3a shows the average correlation across all regions of each subject during the memory stage. The correlation of U‐CapsNet layers (mean *r* = 0.372, 0.418, and 0.389 for L1, L2, and L3, respectively, RSA, Figure 3a) was higher than that of other models (*p* < 10^−10^ with FDR correct, Welsch's *t*‐test, Figure 3a), and the *r*‐values of the Layers 2 and 3 were most significant (*p* < 0.05 with 1000 permutations test in all regions of all subjects, Figure 3a). Therefore, the CAPs in U‐CapsNet accurately explained the brain activation pattern in SWM, and the representations of CAPs in Layer 2 were most similar to those of brain neurons.

We used mediation analysis to explore the relationship between pairs of RDMs (see Section 2). In Figure 3b, the CAPs in Layer 2 were the mediators connecting high‐level spatial features and brain activation (*t* = 16.47, *p* < 10^−10^, Sobel test), and the CAPNs in Layer 2 were the mediators connecting abstract image features and brain activation (*t* = 9.82, *p* < 10^−10^, Sobel test). In addition, all CAPs and CAPNs had complete mediating relationships (the independent variable can solely affect the dependent variable indirectly by means of a mediator). The results suggested that when the brain receives a visual stimulus during a SWM task, the spatial and abstract features of the image may be encoded as a form similar to CAPs and CAPNs, which then form memory. The high‐level CAP in Layer 3 integrated important information from the CAPs in Layer 2, but Layer 3 outcomes explained less brain activation than Layer 2 outcomes, suggesting that Layer 3 has complex nonlinear changes that may be beyond the scope of brain function.

Next, we analyzed the representational similarity between features in U‐CapsNet layers and brain activation under different conditions of trials. The correlations for high memory loads (5 and 7 circles) were significantly higher than those for low memory loads (1 and 3 circles) (*p* < 10^−3^ with FDR correct, Welsch's *t*‐test, Figure 3b). And except for CAPNs in Layer 2, the *r*‐value increased as memory load increased (Figure 3b). This result demonstrated that for images with more complex high‐level spatial features, the representation of brain activation was more similar to that of U‐CapsNet layers.

Moreover, we used an encoding model to compare at the single‐voxel level to map latent representations in computational models to brain voxels. We regarded features from U‐CapsNet and control models as independent variables to predict voxelwise activation. We mainly focused on the cortex closely related to vision and cognition. (The location of these areas was shown in Figure S1.) The average predicted accuracy across voxels in each cortical area was calculated (Figure 3d). The results suggested that the features in U‐CapsNet can better predict brain activation than those in the control models (*p* < .05 with FDR correct, two‐side Welsch's *t*‐test). See Figure S2 for visualization of the distribution and geometry of predicted activations, and Figure S3 for inter‐subject variability of predicted activations.

The CAPs in Layer 2 predicted the activation of frontal region and hippocampus better than other models (*p* < 0.05 with FDR correct, two‐side Welsch's *t*‐test). Surprisingly, although the CAPNs in Layer 2 had relatively low explanatory power for overall brain activation, it can better predict activation of dorsal stream areas, including early visual, lateral occipital, and dorsal (parietal) cortex than other models (*p* < .05 with FDR correct, two‐side Welsch's *t*‐test).

Interestingly, the CAPs and CAPNs of Layer 2 exhibit a complementary phenomenon, where regions accurately explained by the CAPs are not well explained by the CAPNs and vice versa (Figure 3e,f). The results of the regional RSA also support this phenomenon (Figure S4). Our results demonstrated that the CAPs and the CAPNs in Layer 2 explained two different functional mechanisms in the brain. The CAPs more accurately accounted for the activation of high‐level cognitive regions, representing more high‐level spatial features. In contrast, the CAPNs were more similar to activation in the dorsal visual stream regions, which may process abstract visual and spatial features.

### 3.3 Concatenating CAPs explain brain activation during the response stage

After memorizing the image, the participants were shown a probe image and instructed to determine whether the green circle appeared in the correct location. During the response stage, participants not only encoded probe images but also recalled stimulus images. Next, we further analyzed the relationship between features in U‐CapsNet and brain activation during the response stage.

We first fed the probe images into the model trained with stimulus images and obtained the feature maps of each U‐CapsNet layer. To combine the features of two images encoded by U‐CapsNet, we concatenated the features of two images layerwise. Then, we used three classes of features to construct RDM, namely features of stimulus images, features of the probe images, and the concatenation of features of the stimulus and probe images.

**FIGURE 4:**
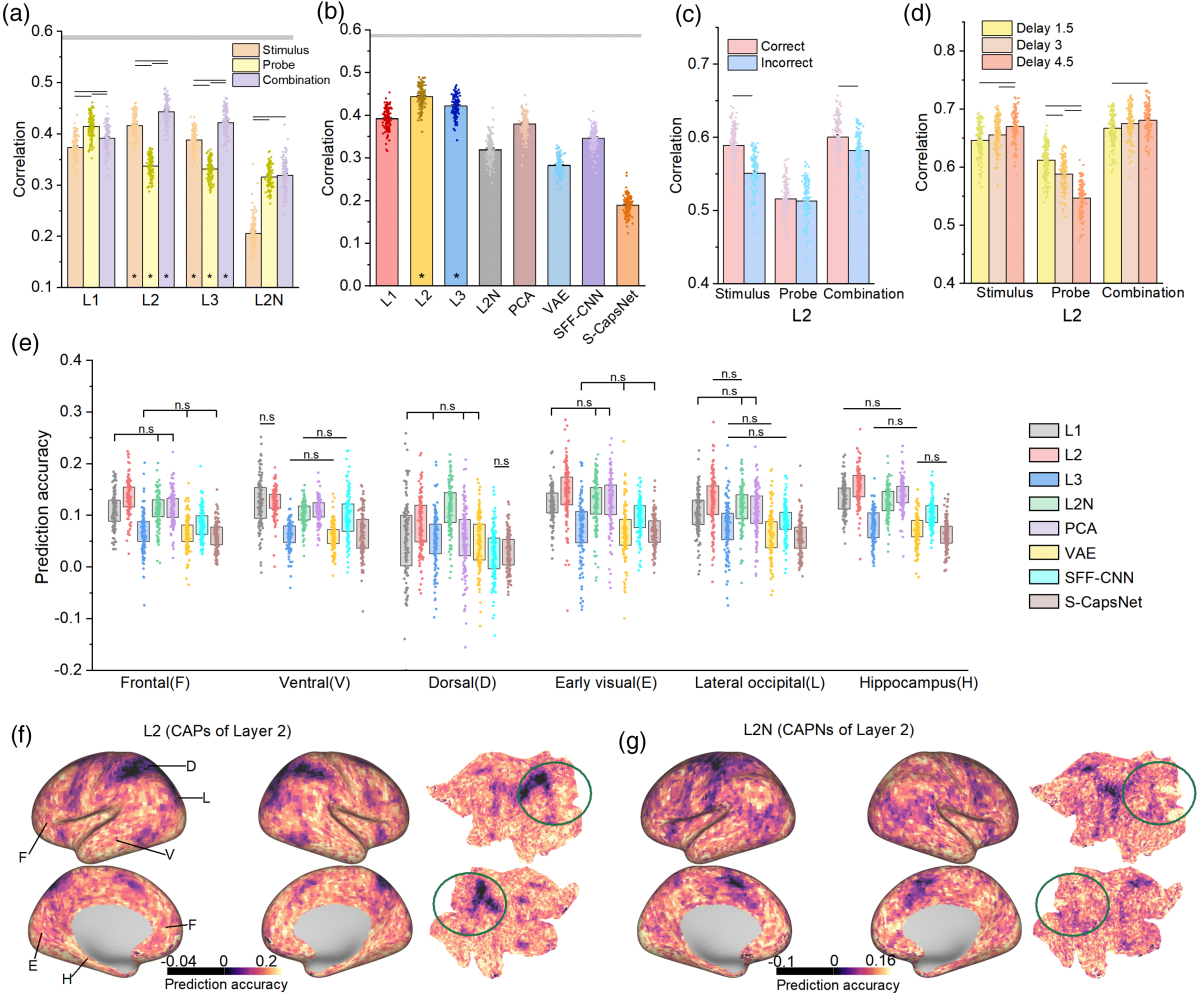
Representational similarity analysis (RSA) between features in U‐CapsNet and brain activation during the response stage. (a) RSA for features of stimulus images, probe images, and combined images, encoded by U‐CapsNet. The gray area indicates a noise ceiling. (b) RSA that using concatenated features from each model. Asterisks (*) indicate significance in all ROIs of all subjects (1000 permutation tests, *p* < .05). (c) and (d) RSA between concatenated CAPs in Layer 2 and brain activation under different conditions. (e) Predicting voxelwise activation using concatenated representations of each computational model. (n.s) indicates no significant differences between pairs of models (*p* < .05 with FDR correct, two‐side Welsch's *t*‐test). The data in the box range from 25% to 75%. (f) and (g) Visualization of prediction accuracy using capsules (CAPs) and capsule neural networks (CAPNs) in Layer 2 (averaged across all subjects). The green circle approximately covers the dorsal visual stream area. The black lines in (a), (c), and (d) indicate significant differences between models. Of note, there were significant differences between any two models in (b). The error bars in (a), (b), (c), and (d) indicate the SE. Asterisks (*) at the bottom of (a) and (b) indicates significance in all ROIs of all subjects (*p* < .05, 1000 permutation test).

For the neurons in Layer 1, the correlations of the probe image were the highest (mean *r* = 0.414; RSA, Figure 4a). For the CAPs in Layers 2 and 3, the correlations of the concatenation of two images were highest (mean *r* = 0.443 and 0.421; RSA, Figure 4a), while the correlations of the probe image were lowest (mean *r* = 0.337 and 0.331; RSA, Figure 4a). This result suggested that the brain mainly encoded the primary visual features in the probe image and the spatial features in the stimulus image during recall and that the brain primarily integrated spatial features rather than visual features of the two images. The probe image consists of a circle containing less spatial information; thus, the brain neurons mainly encode its visual information. When the brain judges whether the green circle appears at the correct position, it may retrieve the spatial features of the stimulus image and compare them with the spatial features of the green circle, and then reconstruct a new overlapping image. Moreover, the concatenated features of U‐CapsNet can better explain brain activation during recall (mean *r* = 0.391, 0.443, and 0.421 for L1, L2, and L3, respectively; RSA, Figure 4b) than those of control models (*p* < 10^−10^ with FDR correct, two‐side Welsch's *t*‐test, Figure 4b). This result further demonstrated the biological plausibility of U‐CapsNet in explaining multiple brain activation patterns.

Next, we calculated the similarity between the RDM of brain activation and concatenated CAPs in Layer 2 under different trial conditions. In Figure 4c, the correlations of stimulus images increased as the delay time increased, whereas the correlations of probe images showed the opposite trend. In Figure 4d, the *r*‐values in the true‐positive trials were significantly higher than those in the true‐negative trials (*p* < 10^−3^, Welsch's *t*‐test). This result further demonstrated that the CAPs accurately distinguished brain activation under different conditions, which was similar to the findings shown in Figure 3b.

Further, we predicted the voxelwise activation during the response stage using the concatenated features (Figure 4e, See Figure S5 for visualization of the geometry of predicted activations, and Figure S6 for inter‐subject variability of predicted activations). The CAPs in Layer 2 better predict activation of frontal and ventral (temporal), early visual, regions and hippocampus (*p* < .05 with FDR correct, two‐side Welsch's *t*‐test, Figure 4e) than other models. While the CAPNs in Layer 2 only better predict the activation of the dorsal region (*p* < .05 with FDR correct, two‐side Welsch's *t*‐test, Figure 4e) than other models. In addition, the complementary effects of the CAPs and CAPNs in the response stage (Figure 4f,g) were less significant than in the memory stage. The regional RSA found similar results (Figure S7). These results suggested that CAP‐like representations may play a dominant role in the brain during the response phase and that the brain is more likely to form CAPN‐like representations during memory than during recall.

### 3.4 The change in representation between CAPs and CAPNs explains the information transfer between brain regions

**FIGURE 5:**
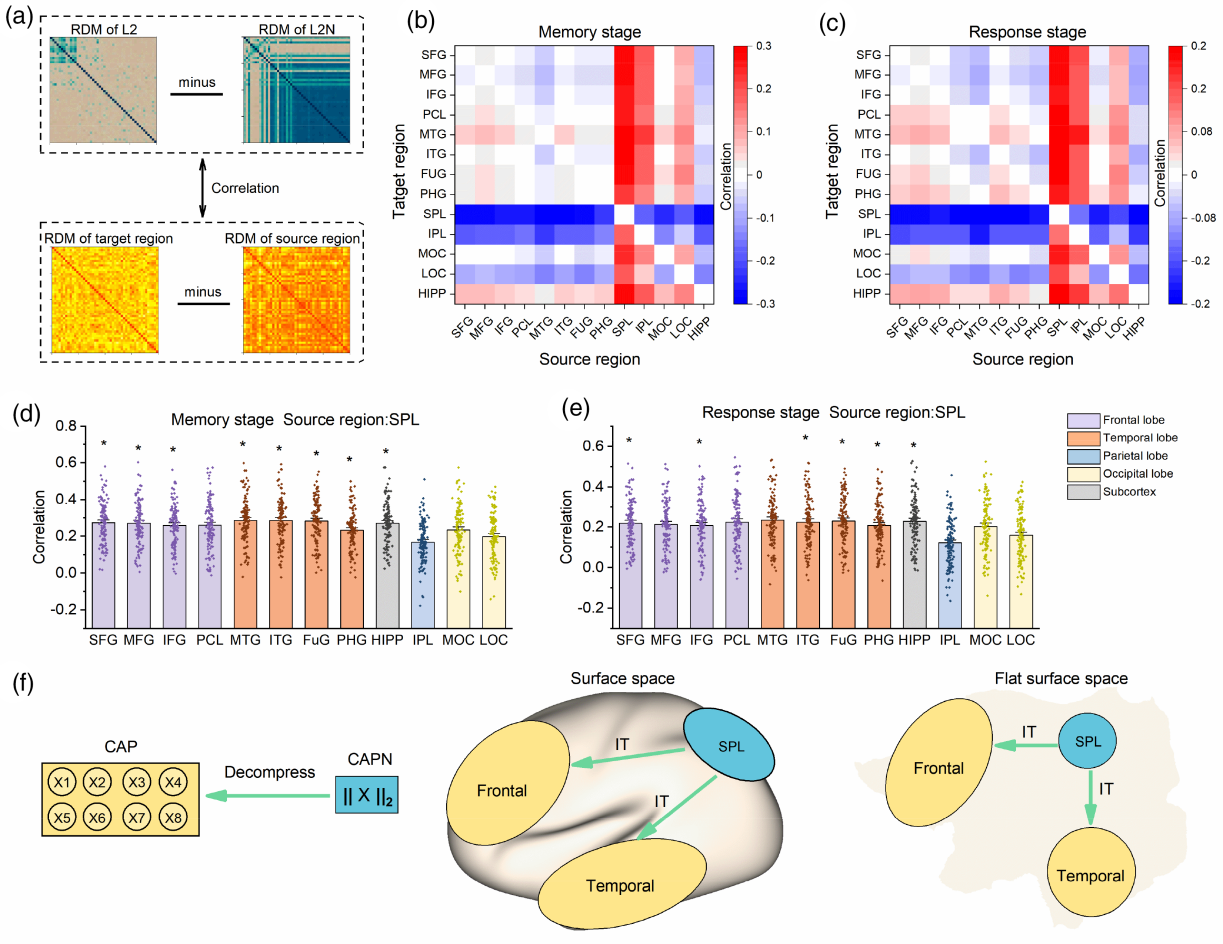
Explainability of the representational difference between capsules (CAPs) and capsule neural networks (CAPNs). (a) Calculating the association between the representational changes using representational similarity analysis (RSA). We first calculated the Z‐score of the representational dissimilarity matrices (RDMs), before performing the minus operation. (b) and (c) The similarity between representational change from CAPs to CAPNs with that of each pair of regions during memory and response stages. (d) and (e) Specific results of the SPL during memory and response stages. Asterisks (*) indicate significance in 90% of subjects (1000 permutation tests, *p* < .05). The error bars indicate the SE. (f) The correspondence between the representations in the U‐CapsNet and the brain. There were CAPN‐like representations in the dorsal stream regions and CAP‐like representations in the frontal and temporal lobes. The linear conversion (decompression) between CAPNs and CAP was similar to the information transfer from the SPL to the frontal and temporal lobes. IT indicates information transfer. FUG, fusiform gyrus; HIPP, hippocampus; IFG, inferior frontal gyrus; IPL, inferior parietal lobule; ITG, inferior temporal gyrus; LOC, lateral occipital cortex; MFG, middle frontal gyrus; MOC, medioventral occipital cortex; MTG, middle temporal gyrus; PCL, paracentral lobule; PHG, parahippocampal gyrus; SFG, superior frontal gyrus; SPL, superior parietal lobule.

The CAPs and CAPNs in Layer 2 represented two levels of image features and explained two classes of brain activation patterns. Next, we explored the physiological significance reflected by the representational changes from the CAPs to the CAPNs using RSA (Figure 5a). We computed the explanatory of representational changes between the CAPs and CAPNs for each pair of inter‐regional representational changes (Figure 5b,c; See Figure S8 for the significance of RSA in all subjects). Specifically, we found that the representational changes from CAPNs to CAPs were significantly associated with the representational changes from the superior parietal cortex to the frontal and temporal regions during both memory and response stages (*p* < 0.05 with 1000 permutations test in 90% subjects, Figure 5d,e). These results suggested that the liner conversion (decompression) between CAPN and CAP was similar to the information transfer from the dorsal stream regions, especially the superior parietal cortex, to the cognitive regions (Figure 5f), enabling the brain to extract high‐level spatial features from abstract features. Thus, U‐CapsNet can also serve as a computational model for the dynamic transfer of high‐level spatial information in the brain.

### 3.5 Relationship between features in U‐CapsNet and human behavior

**FIGURE 6:**
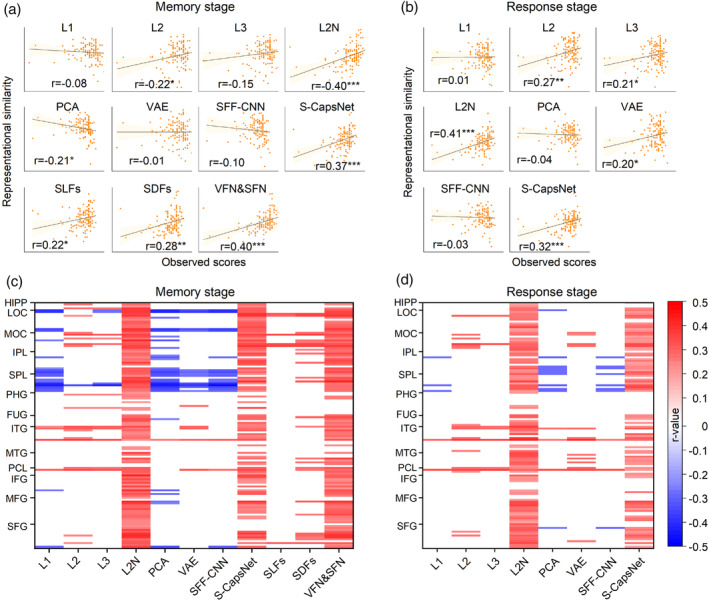
Correlation between representational similarity and memory performance. (a) and (b) Correlation between average representational similarity across ROIs and memory performance for each model during the memory and response stages, respectively. *: *p* < .05; **: *p* < .01, ***: *p* < .001. (c) and (d) Correlation of each ROI during the memory and response stages, respectively. Only significant ROIs are displayed (*p* < .05 with FDR correct, Pearson correlation). The Y‐axis represents ROIs, the X‐axis represents the computational models and high‐level spatial features, and the RIOs were ordered according to brain areas.

Next, we analyzed the relationship between features in U‐CapsNet and human behavior (memory performance calculated from response accuracy). Figure 6a,b show the correlation between the overall representation similarity and memory performance across subjects. (In the response stage, the RDMs of computational models were established by a concatenation of features of the stimulus and probe images.) Figure 6c,d shows the results for ROIs in the BN atlas (Fan et al., 2016) (*p* < .05 with FDR correction, Pearson correlation). Compared with the PCA, SFF‐CNN, VAE, and Layer 1 findings, there were more ROIs showing a significantly positive correlation in Layers 2 (CAPs and CAPNs) and 3. Of note, the distribution of ROIs in SDFs was similar to that in the CAPs of Layer 2 and the distribution of ROIs in VFN & SFN was similar to that in the CAPNs of Layer 2. Thus, the features in Layer 2 were similar to the subjectively defined spatial and abstract features not only in the representational geometry but also in the explanation of brain memory function. To our surprise, we found that almost all ROIs showed a significant correlation for the CAPNs and VFN & SFN. Moreover, the similarity between the CAPNs and brain activation had a significant correlation with matrix reasoning performance of humans that forms the basis of learning ability (*p* = 0.017, Pearson correlation; Table S5). This result demonstrated that the CAPN‐like representations built into brain neurons, which process abstract features, are essential for the formation of SWM. Almost all regions in the superior parietal cortex showed negative correlations in models representing primary visual features, such as features in Layer 1, PCA, VAE, and SFF‐CNN, and positive correlations in models representing abstract features, such as CAPNs, VFN & SFN, and features in S‐CapsNet. Together, these findings demonstrate that features of U‐CapsNet can explain human behavior well and accurately reflect the contribution of regions to memory function.

## 4 DISCUSSION

U‐CapsNet overcomes some limitations (Doerig et al., 2019; Geirhos et al., 2018; Kietzmann et al., 2019; Yamins & DiCarlo, 2016) in previous SFF‐CNN models and has better biological plausibility to capture neural mechanisms. In this study, we used a U‐CapsNet as a computational model of spatial representation for the human brain. We suggested that the representational spaces in the U‐CapsNet spontaneously extracted some human‐defined spatial features. Importantly, we provided neural evidence that representational spaces in U‐CapsNet capture the brain response structures during recognizing and recalling spatial information, as well as key factors affecting human behavior. In addition, our results make it possible to reveal the abstract spatial representation in the dorsal stream region, which plays a dominant role in processing spatial information.

Our results corroborate and extend a previous report that the combination of unsupervised learning and recurrent architectures is the most suitable method for exploring brain mechanisms (Zhuang et al., 2021).CapsNet was originally designed to develop a DNN with more computational properties of the brain's nervous system (Alom et al., 2019; Sabour et al., 2017), facilitating extract global features and take into account the relative relationships between specific features (Alom et al., 2019, Sabour et al., 2017). Our study shows that CapsNet can capture hand‐drawn spatial features while PCA, VAE, and SFF‐CNN mainly focused on low‐level visual features, reinforcing the view that humans and CapsNet may understand space in a similar way. Furthermore, the dynamic routing mechanism integrates important information through its unique recurrent processing. We propose that its nonlinear computation is comparable to feedback mechanisms in the visual system, creating a brain‐like representational schema. Unsupervised learning is superior to supervised learning when employing CapsNet for brain modeling, suggesting human visual system does not rely on tutored statistical learning but instead leverages simple proxy tasks to prompt formation of interpretable spatial representation. Indeed, the biological feasibility of unsupervised learning has been demonstrated in theory and practice (Higgins et al., 2021; Lindsay, 2021; Seung & Lee, 2000). Our work extends the application of unsupervised learning, reporting a finding that the brain uses unsupervised learning not only to encode sensory signals into the semantically meaningful representations in object identification tasks but also to conclude the storable and retrievable high‐level context of targets in cognitive tasks. Overall，employing the appropriate DNN architecture and learning strategy, based on the functional properties of the brain, is crucial for comprehending the brain's visual processing.

Our proposed U‐CapsNet was demonstrated by the overall results to contain the inductive basis that is needed to build an effective high‐performing but biologically plausible visual learning system (Zador, 2019). From the neurobiological perspective, our U‐CapsNet accurately explained and predicted fMRI activation, outperforming the control methods. In detail, the CAPs in Layer 2 can commendably explain the activation in higher cognitive regions but not that in dorsal stream regions. The frontal region is important for executive attention and the maintenance of spatial information (Kane & Engle, 2002; Kastner et al., 2007; Zimmer, 2008). The hippocampus is closely associated with recall and imagery (Gelbard‐Sagiv et al., 2008). The temporal region is important for facial and semantic cognition (Davey et al., 2016). These results suggested that the frontal and temporal lobes have functionality that enables them to evaluate statistical data similar to that of CAPs, so it can be used to integrate and maintain spatial features, forming short‐term memory (Curtis, 2006; Thomas et al., 1999; Zimmer, 2008). A recent study showed that both the primate prefrontal lobe and corresponding recurrent neural network model utilize units with long timescales to sustain WM information (Kim & Sejnowski, 2021). Therefore, DNNs have great potential to model various mechanisms in the higher cognitive regions.

A CAPN represents the probability of the existence of a specific feature, and it can also be viewed as a further compression or integration of features. In this study, the CAPNs significantly encoded abstract information of visual and spatial features and better explained and predicted activation of dorsal stream regions than that of frontal and temporal regions， and the change in representation from CAPNs to CAP explained the information transfer from dorsal stream regions to frontal and temporal regions. The dorsal visual stream, transferring information to the posterior parietal cortex, is mainly involved in spatial position cognition (Goodale & Milner, 1992). The lateral occipital cortex plays a role in visual search, and the superior parietal cortex is crucial for representing the position of the target (Silk et al., 2010; Silver et al., 2005; Zimmer, 2008). Evidence have shown that substantial compression and nonlinear processing of visual signals occurs before the cerebral cortex (Gollisch & Meister, 2010; Hayhoe & Ballard, 2005; Hosoya et al., 2005). The interactions between dorsal stream regions and the frontal and temporal lobes are crucial for working memory (Ghuman et al., 2008; Mechelli et al., 2004; Sakreida et al., 2016). Thus, we discovered a significant resemblance between the intermediary stage of the visual system and the intermediate layer of U‐CapsNet, which may explain a pathway of information processing in the brain during SWM tasks. U‐CapsNet first processes the signal primitively, then extracts the properties and probability of existence of each entity, and finally uses a recurrent process to select the important features for decoding. In brain, the preprocessed image first enters the primary visual cortex and is then encoded into CAPN‐like representations along the dorsal stream. Finally, the superior parietal cortex transfers most of the encoded information to the frontal and temporal lobes, where it is completely decompressed into CAP‐like representations and stored as memory. Of note, for almost all ROIs, the similarity between regional activation and CAPNs greatly determines memory performance. This demonstrates that CAPN‐like representations may be basic metabolites in each neuron during SWM and that the representation of abstract features may be the most basic spatial feature easily recognized by the brain system.

The superior parietal cortex plays a crucial role in representing a spatial map or coordinates and in allocating spatial attention, which is important for encoding and retrieving spatial locations (Ayzenberg & Behrmann, 2022; Silk et al., 2010; Silver et al., 2005; Zimmer, 2008). Using U‐CapsNet to mimic the brain to perform SWM tasks, our study further explains the importance of the superior parietal cortex. We demonstrate that the superior parietal cortex is particularly sensitive to representations of abstract information rather than that of subjectively defined spatial features. The representation of primary visual information even disrupts the function of the superior parietal cortex. Moreover, the information transfer from the superior parietal cortex to the frontal and parietal regions is essential for the extraction of high‐level spatial features. These results suggested that the superior parietal cortex is a hub region with specific functions in SWM, the main purpose of which is to connect the preceding and the following. It acts as an adapter and filters in the middle of the process, changing the format of the representation and allowing only such format features to enter subsequent processors. In some complex computer vision tasks, the activation pattern of the superior parietal cortex may serve as a supervision to regulate the semantic information in the model, leading to brain‐like DNNs that enhances reasoning ability.

Brain activation in the response stage can be accurately explained by concatenating encoded features of stimulus and probe images. This suggests that the brain does retrieve the spatial features of stimulus images during recall from the perspective of the DNN. This finding also corroborates a previous report that imagery and perception share common neural mechanisms (Dijkstra et al., 2019; Xie et al., 2020) and demonstrated that the mechanisms used in the brain to encode high‐level spatial features of both stimulus and probe images act as the calculation principle in U‐CapsNet at its core. When the brain determines whether the circle is in the target position, it may not perform a further complex fusion of the encoded spatial features of the two images but may simply linearly combine them to reconstruct the scene where the two images overlap.

Furthermore, we believe that U‐CapsNet performed each trial in SWM perfectly because its high‐level CAP can be used to reconstruct images accurately. For humans, different types of trials have different levels of complexity, so the brain activates differently under various conditions. Whether we divided the types of trials according to the number of circles, delay time, or the correctness of position, the features in U‐CapsNet could explain various types of brain activation with different accuracies. In addition, the similarity between U‐CapsNet representations and fMRI response had a dominant effect on individual memory performance. These results demonstrate that U‐CapsNet could be used to evaluate the brain's perception of task complexity and predict the brain's memory ability. Therefore, activation in U‐CapsNet can be regarded as the “gold standard,” and the distance between brain activation and the “standard” has obvious physiological significance.

In this study, we did not find gradient mapping. Some previous studies have also shown this phenomenon (Cross et al., 2021; St‐Yves et al., 2022). This outcome in the present study may be due to the architecture of the autoencoder model. The learning objective for autoencoding is considered as “free energy” minimization, which is a principle of the brain mechanism (Friston, 2010). Free energy should be defined for every level of the visual hierarchy, whereas the autoencoder only minimizes the free energy concerning the input level and a single‐level latent space (Han et al., 2019). In addition, the experimental paradigm of SWM was relatively simple, which may hinder the training of a high‐performance DNN, and the memory and response tasks were performed simultaneously, which may affect the accuracy of the estimation of brain activation in both stages. In the future, we can design more reasonable experimental paradigms to deeply and comprehensively explore the consistency between DNNs and the brain.

## 5 CONCLUSIONS

U‐CapsNet has better biological plausibility than SFF‐CNNs in terms of computational principles. We analyzed the similarities between U‐CapsNet representations and brain representations from several aspect by instructing U‐CapsNet and humans perform the same SWM task. Our results demonstrate that U‐CapsNet more accurately explains brain activation and human behavior. This study not only provided a computationally feasible framework for modeling how the human brain encodes spatial features but also provided insights into the representational format and goals of the human brain. Our results suggested that a synergy between U‐CapsNet and cognitive neuroscience offers the promising prospect for a deep and comprehensive understanding of the internal representations of intelligent systems.

## CONFLICT OF INTEREST STATEMENT

The authors declare no competing financial interests.

## Supporting information


**DATA S1:** Supporting Information.

## Data Availability

The raw fMRI and MRI data are publicly available at https://legacy.openfmri.org/dataset/ds000030/. The code of U‐CapsNet is publicly available at https://github.com/book-book24/U-CapsNet. Any additional information required to reanalyze the data reported in this paper is available from the lead contact upon request.
