# Supplementary material for: Using unsupervised capsule neural network reveal spatial representations in the human brain
Source: Hum Brain Mapp. 2024 Mar 27;45(5):e26573. doi: 10.1002/hbm.26573 (PMC10973701; doi:10.1002/hbm.26573)
Supplement: Supplementary file 1 — DATA S1: Supporting Information. [file HBM-45-e26573-s001.docx]

**Supplementary information**

**
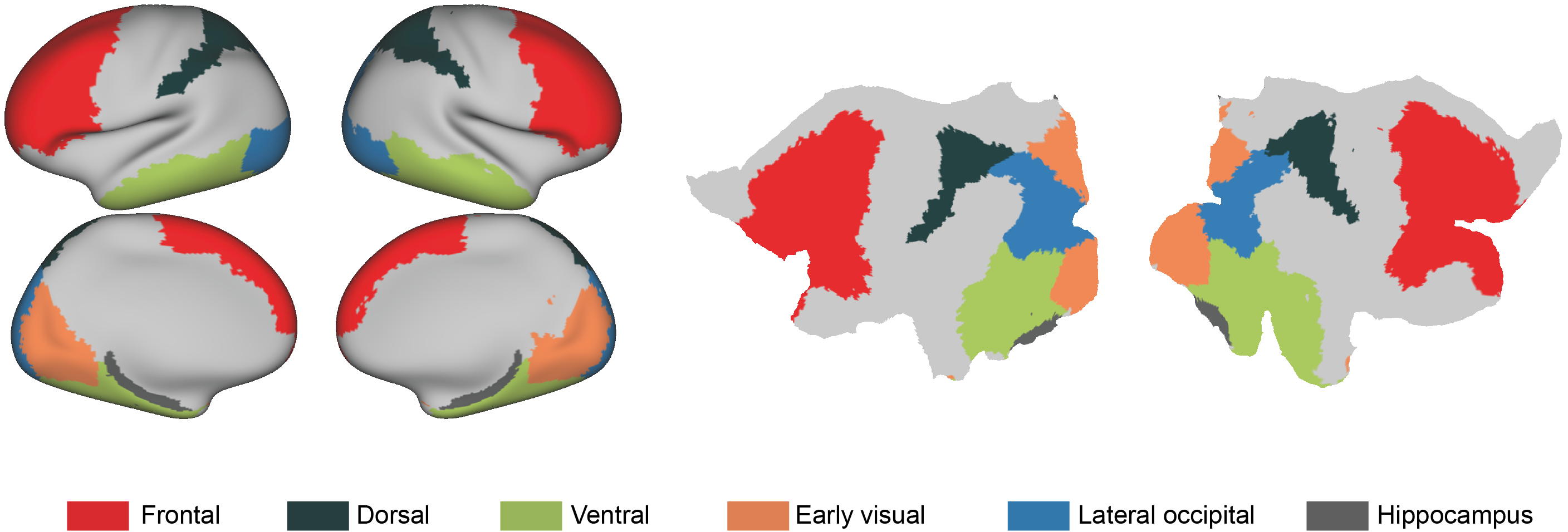
**

**Fig. S1. Visualize the regions of interest for this study.** These regions are closely related to spatial working memory.



 **Fig S2.** **Predicted regional activation in memory stage visualized in a 2-D space obtained with t-SNE.**

**

**

**Fig. S3. Intersubject variability of predicted activation during memory stage.** It was quantified by averaging the correlation values between the activation series of any two subjects.Intersubject variability was calculated for each voxel. **(a)** Average intersubject variability across voxels of predicted activations. (n.s) indicates no significant differences between pairs of models. The error bars indicate the SE. (**b)** Intersubject variability of predicted regional activations (Intersubject variability of voxels within a region were averaged). ‘Actual’ indicates the intersubject variability of actual activation.

**

**

**Fig. S4.** **RSA between features in U-CapsNet and regional activation during the memory stage.** RSA between CAPs and CAPNs with activation of each area, respectively. Asterisks (*) indicate significance in all subjects (1000 permutation tests, p<0.05). Gray areas indicate noise ceilings. The dotted lines indicate average correlation across all ROIs. The error bars indicate the SE. HIPP: hippocampus, SFG: superior frontal gyrus, MFG: middle frontal gyrus, IFG: inferior frontal gyrus, PCL: paracentral lobule, FUG: fusiform gyrus, MTG: middle temporal gyrus, ITG: inferior temporal gyrus, PHG: parahippocampal gyrus, IPL: inferior parietal lobule, SPL: superior parietal lobule, MOC: medioventral occipital cortex, LOC: lateral occipital cortex.

**

**

**Fig. S5.** **Predicted regional activation in response stage visualized in a 2-D space obtained with t-SNE.**

**

**

**Fig. S6. Intersubject variability of predicted activation during response stage. (a)** Average intersubject variability across voxels of predicted activations. **(b)** Intersubject variability of predicted regional activations (Intersubject variability of voxels within a region were averaged). ‘Actual’ indicates the intersubject variability of actual activation.





**Fig. S7.** **RSA between features in U-CapsNet and brain regional activation during the response stage.**RSA between concatenated CAPs and CAPNs with activation of each area. Asterisks (*) indicate significance in all subjects (1000 permutation tests, p<0.05). Gray areas indicate noise ceilings. The dotted line indicates the average correlation across all ROIs.





**Fig. S8. Significance of the explanatory representational changes between CAPs and CAPNs.** The values in the heat map indicate what proportion of subjects (n = 129) exhibit significant similarity (1000 permutation tests, p<0.05).

**Table S1. Parameter settings of U-CapsNet**

| **Network architecture** | **Encoder** | | | **Encoder to Decoder** | **Decoder** | | |
| --- | --- | --- | --- | --- | --- | --- | --- |
|  | Convolutional Layers | Primary Capsule Layer | Higher Capsule Layer | Fully connected layer | Deconvolution layer + LeakyReLU | Deconvolution layer + LeakyReLU | Deconvolution layer + LeakyReLU |
| **Parameter setting** | kernel size = 9×9 filters = 64 stride = 1 | kernel size = 9×9, filters = 256,  stride = 2,  capsules size = 10, | capsules size = 40, routing iteration = 3 | dimension = 1024 | kernel size = 9×9,  filters = 64, stride = 1, padding =1 | kernel size = 9×9,  filters = 256, stride = 1, padding =1 | kernel size = 4×4,  filters = 3, stride = 1, |

**Table S2. Parameter settings of VAE**

| **Network architecture** | **Encoder** | | | **Encoder to Decoder** | **Decoder** | | |
| --- | --- | --- | --- | --- | --- | --- | --- |
|  | Convolutional Layer + Relu | Convolutional Layer + Relu | Convolutional Layer + Relu | Fully connected layer | Deconvolution layer + LeakyReLU | Deconvolution layer + LeakyReLU | Deconvolution layer + LeakyReLU |
| **Parameter setting** | kernel size = 9×9,  filters = 256, stride = 1 | kernel size = 3×3, filters = 64,  stride = 1, padding = 1 | kernel size = 3×3, filters = 64,  stride = 1, padding = 1 | dimension of mean = 40, dimension of variance = 40 | kernel size = 3×3, filters = 64,  stride = 1, padding =1 | kernel size = 3×3, filters = 64,  stride = 1,  padding =1 | kernel size = 9×9,  filters = 256, stride = 1 |

**Table S3. Parameter settings of SFF-CNN**

| **Network architecture** | **Encoder** | | | **Classifier** |
| --- | --- | --- | --- | --- |
|  | Convolutional Layer + Relu | Convolutional Layer + Relu | Convolutional Layer + Relu | Fully connected layer + softmax |
| **Parameter setting** | kernel size = 3×3,  filters = 64,  stride = 1 | kernel size = 3×3,  filters = 128,  stride = 1 | kernel size = 3×3,  filters = 256,  stride = 2 | number of classes = 4 |

**Table S4. Parameter settings of S-CapsNet**

| **Network architecture** | **Encoder** | | | **Classifier** |
| --- | --- | --- | --- | --- |
|  | Convolutional Layers | Primary Capsule Layer | Higher Capsule Layer | Fully connected layer + softmax |
| **Parameter setting** | kernel size = 9×9 filters = 64 stride = 1 | kernel size = 9×9, filters = 256, stride = 2, capsules size = 10, | capsules size = 40, routing iteration = 3 | number of classes = 4 |

**Table S5. Correlation between representational similarity and matrix reasoning ability.**

| Model | age | |  | Response stage | |
| --- | --- | --- | --- | --- | --- |
|  | r-value | p-value |  | r-value | p-value |
| L1 | -0.060 | 0.510 |  | 0.010 | 0.917 |
| L2 | 0.094 | 0.304 |  | 0.114 | 0.211 |
| L3 | 0.053 | 0.566 |  | 0.098 | 0.286 |
| L2N | **0.217** | **0.017*** |  | 0.174 | 0.056 |
| PCA | -0.140 | 0.126 |  | -0.010 | 0.913 |
| VAE | -0.101 | 0.271 |  | 0.155 | 0.089 |
| SFF-CNN | -0.097 | 0.289 |  | -0.017 | 0.852 |
| S-CapsNet | **0.241** | **0.008*** |  | 0.178 | 0.050 |
| SLFs | 0.097 | 0.290 |  |  |  |
| SDFs | 0.147 | 0.106 |  |  |  |
| VFN&SFN | **0.201** | **0.027*** |  |  |  |
